# Cancer metastasis: enactment of the script for human reproductive drama

**DOI:** 10.1186/s12935-017-0421-y

**Published:** 2017-05-02

**Authors:** Xichun Sun, Xiwu Liu

**Affiliations:** 1 Department of Pathology and Laboratory Medicine, McGuire Holmes Veteran Affairs Medical Center, School of Medicine, Virginia Commonwealth University, 1201 Broad Rock Boulevard, Richmond, VA 23249 USA; 2 Department of Hepatobiliary Surgery, People’s Hospital of Hunan Province, Hunan, China

## Abstract

Based on compelling evidence from many biological disciplines, we put forth a hypothesis for cancer metastasis. In the hypothesis, the metastatic cascade is depicted as human reproduction in miniature. Illustrated in a reproductive light, the staggering resemblance of cancer metastasis to human reproduction becomes evident despite some ostensible dis-similarities. In parallel to the appearance of primordial germ cells during early embryogenesis, the cancer reproductive saga starts with the separation of metastasis initiating cells (MICs) from cancer initiating cells when the primary cancer is still in its infancy. Prime MICs embark on a journey to the host bone marrow where they undergo further development and regulation. Migrating MICs are guided by the same CXCR4/CYCL12 axis as used in the migration of primordial germ cells to the genital ridge. Like the ovary, the host bone marrow features immune privileges, coolness, hypoxia and acidity which are essential for stemness maintenance and regulation. Opportune activation of the MICs via fusion with bone marrow stem cells triggers a frenzy of cellular proliferation and sets them on the move again. This scenario is akin to oocyte fertilization in the Fallopian tube and its subsequent journey towards the decidum. Just as the human reproductive process is plagued with undesirable outcomes so is the cancer metastasis highly inefficient. The climax of the cancer metastatic drama (colonization) is reached when proliferating MIC clusters attempt to settle down on decidum-like premetastatic sites. Successfully colonized clusters blossom into overt macrometastases only after the execution of sophisticated immunomodulation, angiogenesis and vascular remodeling. Similarly, the implanted blastomere needs to orchestrate these feats before flourishing into a new life. What is more, the cancer reproductive drama seems to be directed by a primordial hypothalamus–pituitary–gonad axis. Pursuing this reproductive trail could lead to new frontiers and breakthroughs in cancer research and therapeutics.

## Background

Despite decades of global efforts, cancer still remains one of the major human killers. It is estimated that more than 90% of cancer related deaths are due to metastases rather primary cancers [1]. Whereas our trump cards in the current anticancer arsenal indeed show impressive effects in treating primary cancers, effective anti-metastasis therapeutics remain elusive. To make things worse, evidence exists that most of the cancer treatment modalities might promote metastasis, tumor resistance and relapse [2–5].

The lack of effective anti-metastasis regimens largely stems from our rudimentary understanding of cancer metastasis. The linear and parallel hypotheses represent the two prevailing metastasis theories [6]. They converge on a multistep process and an evolutionary theme, but differ on the timeframe when it occurs in reference to the ontogeny of the primary tumor. The former stipulates that metastasis occurs at a late stage when a fittest sub-clone develops as a result of selection pressure. The latter argues for an early metastatic event and parallel evolution of the primary and metastatic cancer.

Reproduction is fundamentally and biologically what all the life on this planet is about regardless of its form and complexity. Emerging lines of evidence suggest that cancer metastasis represents the enactment of the powerful human reproductive blueprint (script) which is fashioned out by Mother Nature through four billion years of incessant biological warfare and two billion years of sexual reproduction experience on this ever- evolving planet. Despite some variations, cancer metastasis can be depicted as human reproduction in miniature. In accordance with the parallel theory, we put forth this hypothesis of cancer metastasis in hopes of furthering cancer research and expediting the development of efficacious anti-metastasis therapeutics.

## Metastatic drama portrayed in a reproductive light

### Metastasis initiation entails relative cellular DNA integrity

The Darwinian linear model of cancer metastasis has long been questioned by leading cancer researchers. As the secrecy over the complexity and sophistication of cancer metastasis starts to be unraveled, it becomes increasingly difficult to contemplate that cells riddled with DNA abnormalities would be able to pull it off. For instance, epithelial–mesenchymal transition (EMT) and mesenchymal–epithelial transition (MET) are two interchangeable morphological processes essential for the completion of the metastasis cascade. Morphologic somersaults of this proportion demand genomic integrity [6, 7]. On the same note, successful initiation of metastasis and metastatic growth depend on exquisite and timely manipulation of the host immune apparatus in a manner similar to that in human pregnancy. Studies in the reproductive medicine field reveal that most human pregnancies end up as unnoticed miscarriages as a result of defective DNA on the part of the embryo [8]. In clinically overt early abortions, an association with the embryonal and/or parental chromosomal abnormalities has been firmly established [9, 10]. Improper antigen expression on the part of an implanting blastomere renders it easy prey for maternal NK cells [11]. Even minor mutations affecting fetal polymorphisms on HLA G, HLA-C and Beta HCG predispose the conceptus to rejection by the uterus [12, 13].

Metastasis initiating cell (MICs) represent prime stem cells with much DNA integrity [14]. Large scale genome sequencing studies of metastatic cancers unravel only enrichment of classical initiator oncogenes rather than metastasis driver genes. In several cancer types, the metastatic tumor could be traced back to a small fraction of cells in the periphery of the primary cancer which contain much less DNA abnormalities than do the bulk of primary cancer cells [15, 16]. Along the same line, disseminated cancer cells in the bone marrow display significantly less genomic aberrations than do their counterparts in the primary cancer before overt metastasis manifests [17].

### MICs are set aside by instinct during primary cancer initiation

It has recently come to light that primary cancer and metastasis initiation probably involve a similar process and further mutations are unnecessary for the latter [18, 19]. Ample evidence indicates that the dissemination of cancer cells to the bone marrow starts very early in the cancer ontogeny [20]. For most primary cancers, tumor initiation requires only a few mutations to revive the embryonic transcription network [21]. Most of the mutations and other genetic abnormalities detected in cancer are incurred from collateral damage during its ontogeny. This notion of setting aside prime MICs from cancer initiating cells (CICs), and then sending them off to the bone marrow dovetails nicely with the phenomenon of early selection of primordial germ cells from the totipotent embryonic cells and their subsequent migration to the genital ridge. It is therefore postulated that MICs are selected and dispatched to the bone marrow during the initiation of primary cancer as an act of reproductive instinct. They are unlikely the product of random mutations acquired through a Darwinian selection as stipulated in the linear hypothesis.

### Epigenetic regulation underpins metastasis

Just as successful human reproduction hinges on well- coordinated epigenetic regulation, so do metastasis and primary cancer initiation invoke the epigenetic machinery [22–24]. Many metastasis specific methylation and microRNA signatures have been identified. For example, epigenetic regulation of *Kisspeptin*-*1 (KISS1) and NM23* (two important metastasis suppressor genes) rather than their mutations are found to be instrumental in many steps of the metastatic cascade [25, 26]. Their over-expression followed by down-regulation seems to be essential for successful colonization of MICs and subsequent metastatic growth respectively just as is it for the proper implantation of the blastocyst and the following placentation [27, 28].

### Evidence of a primordial hypothalamus–pituitary–gonad (HPG) axis in cancer and its link to metastasis (or prognosis)

**Fig. 1:**
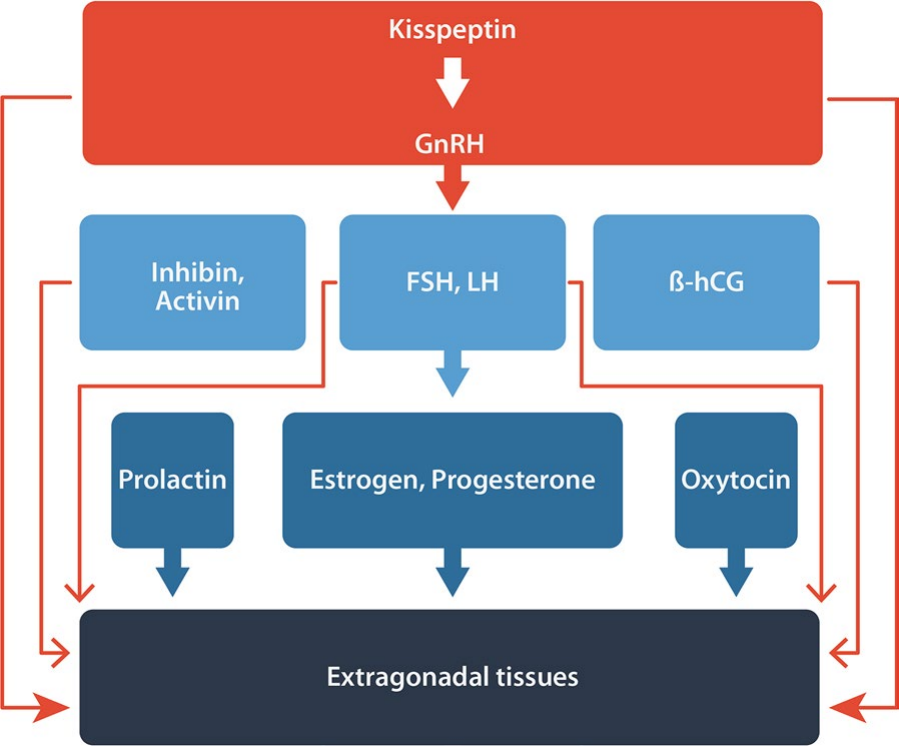
A simplistic version of the expanded HPG axis in Human and cancer reproduction. The axis is expanded to include prolactin, oxytocin and the activing-inhibin axis as well as non-traditional actions such as the extrapituitary actions for GnRH and extragonadal actions for FSH and LH. Complex feedback and interactions among the axis members are omitted for simplicity. *HPG* hypothalamus–pituitary–gonad, *GnRH* gonadotropin releasing hormone, *FSH* follicle stimulating hormone, *LH* luteinizing hormone, *HCG* human chorionic glycoprotein

Ever accumulating evidence suggests that there exists a primordial HPG- like axis in cancer. Figure 1 is a simplified, yet more inclusive version of the HPG axis shared by both human and cancer reproduction. Firstly, Kisspeptin-1 (KiSS1) and its G protein coupled receptor GPR54 are widely present in cancer and their involvement in both carcinogenesis and metastasis has been intensively documented [29, 30].

Gonadotropin-releasing hormone (GnRH) and its receptor have been reported in many cancers from non-reproductive organs [31, 32]. Similarly, the expression of glycoprotein hormones (LH/HCG, FSH) and/or their receptors are widespread in human malignancies [33–35].

Not only have sex hormones been increasingly incriminated in the carcinogenesis of non-gynecological malignancies such as lung, colon, bladder and thyroid cancer, but their secretion and receptor expression are also reported across the cancer spectrum [36–38]. What is more, pregnancy is known to facilitate cancer progression and many pregnancy- related hormones have been linked to metastasis [39–42]. For instance, serum levels of pregnancy hormones such as beta human chorionic gonadotropin (HCG), pregnancy associated protein A (PAP-A), placenta growth factor (PGF), and estrogen correlate well with cancer progression. Even progesterone induced blocking factor (PIBF) has been reported in a variety of malignancies. Unsurprisingly, sex hormone receptor antagonists such as (Ru486) as well as receptor inhibitors for other hormones in the axis have shown efficacy in treating various non-gynecological cancers [43].

Substantial evidence also links oxytocin, prolactin and their receptors to carcinogenesis and cancer progression as well [44, 45]. As an activin/inhibin/follistatin system plays an indispensable role in female reproduction so does it seem to operate in cancer progression [46, 47].

### Host bone marrow: surrogate gonad for cancer

The host bone marrow occupies a unique place in the procreation efforts of cancer [48]. Not only does it offer a safe haven (low temperature, acidity, hypoxia and immune privilege) for MICs, but it also possesses the infrastructure for complex stem cell regulation and integration of information on host’s nutritional, immune and neuroendocrine status. A close developmental link has been recently established between the estrogen and progesterone receptor (ER, PR) expressing hematopoietic stem cells and germ line cells [49, 50]. The fact that epithelial cancers are capable of producing erythroid precursors and expressing erythropoietin receptors putatively places the MICs (and CICs) at least on par with the pluripotent embryonal stem cells (one rank above the hematopoietic stem cells in the stem cell echelon) [51, 52]. As a matter of fact, an embryonic stem cell- like gene signature has been identified in poorly differentiated cancers [53]. Thus, MICs probably express estrogen receptor (ER), progesterone receptor (PR), placental alkaline phosphatase (PLAP), and erythropoietin receptor and employ the same CXCR4/CYCL12 axis in guiding their migration from the primary cancer to the host bone marrow as in the trafficking of the hematopoietic stems and primordial germ cells [54].

Disseminated cancer cells in the bone marrow nevertheless represent a dynamic, heterogeneous group [17]. Before the manifestation of metastasis, the residing cells display genetic immaturity with much genomic integrity suggesting that most of them are likely MICs or their direct descendants. With overt metastasis, the population is replaced by less advantageous cells which have apparently undergone proliferation, selection probably in the metastatic site and/or primary site. At this time, the majority of the population is probably composed of non-stem cells. These cells might still retain the capabilities to undergo proliferation, mobilize and even colonize at distant organs. They, however, lack the potential to bring forth overt metastases just as migrating and tissue trophoblasts in microchimerism fail to blossom into an embryo or placenta [55].

#### Activation of MICs in bone marrow

Whereas the parthenogenetic theory is currently favored for both cancer and metastasis initiation, emerging evidence points to the possibility of a quasi-sexual mode in which the activation of MICs is triggered via cell fusion in a manner akin to fertilization in the Fallopian tube. One candidate activator is the ER+ , PR+ bone marrow mesenchymal stem cells [49]. Alternatively, the bone marrow monoblasts might provide the impetus [6].

Upon activation, MICs start a frenzy of cell proliferation and are set in motion again. This scenario bears astonishing resemblance to the migration of a rapidly proliferating blastomere from the Fallopian tube to the de-cidualized uterine wall and illustrates an important biological tenet: there is safety in numbers. This time the destination is the pre-determined distant tissues (pre-metastatic sites). Abundant evidence indicate that cancer cell clusters have much better chances of surviving the grueling transit and colonization processes than do single cancer cells [56, 57]. The mobilization and trafficking of MICs in the bone marrow may involve the same omnipresent CXCR4/CXCL12 axis.

### Colonization of MIC clusters at premetastatic sites and flourishing into marcometastases

#### Colonization at premetastatic sites

The preparation of premetastatic sites is largely orchestrated by the primary cancer via the secretion of cancer tumor—derived secreted factors and recruitment of bone marrow derived cells [58, 59]. While no direct link has yet been made to pregnancy related hormones, the sites closely resemble the decidua in that both feature a hypoxic, proinflammatory milieu rich in adhesion and survival molecules. As such a milieu is essential for preimplantation incubation, adhesion and implantation of an arriving blastomere, so would it be for the successful colonization of proliferating MIC clusters as evidenced in primary cancer initiation which requires a similar Th1 predominant inflammatory microenvironment featuring over-expression of MHC molecules and presence of CD56 bright NK cells [60].

#### Metastatic dormancy: analogy to intrauterine growth restriction or even diapause

Metastasis characteristically manifests long dormancy which might be viewed as a protracted version of intrauterine growth restriction. Dormancy at the metastatic sites is thought to arise as a result of either insufficient angiogenesis or immune inhibition following colonization.

Nevertheless, it is intriguing to contemplate the possibility that metastatic dormancy might also happen prior to colonization. Diapause is a common reproductive phenomenon which has been observed in many vertebrates including mammals [61, 62]. In diapause, an arriving embryo could hold off implantation for a considerably long period of time until ambient nutrients become abundant. More importantly, it can be induced in species which don’t exhibit such phenomenon naturally. Whereas the exact mechanisms underlying diapause and metastatic dormancy are still unclear, both appear to employ a same set of molecules in pulling off this time-game [63–66].

#### Adequate immunomodulation: prerequisite to macrometastasis

Just as a transition to an anti-inflammatory environment (Th2) is essential for subsequent embryonal development and growth, so would it be for the flourishing of colonized MIC clusters. In keeping with it are studies showing a similar transition in tumor progression from carcinoma in situ to invasive cancer [67]. This immune switch seems to be as sweeping as the one manifesting at the embryo-maternal interface [68, 69].

### Placentation: common theme in both primary and metastatic cancer

#### Cancer-host interface: analogue of a primordial placenta

Accumulating evidence suggests that the interface between primary cancer and the host tissue might be viewed as an analogue of the primordial placenta. Firstly, primary cancer expresses a slew of trophoblastic markers with many of them being restricted to this interface [35, 70–74]. The expression of some of the markers has been linked with cancer progression and prognosis. In analogous to the positioning of NK cells at the embryo-maternal interface, tumor infiltrative NK cells are also restricted largely to this region. Similarly, this shell- like region possesses a vasculature which expresses FSH receptors, produces progesterone and features the countercurrent mechanism all of which are characteristics of a placenta [34, 75]. More importantly, the feeder vessels of the primary cancer and the uterine spiral arteries (vessels feeding the placenta) are both remodeled into conduit- like structures [76, 77].

Lastly, primary cancer and the placenta seem to employ a similar invasion mechanism(s) to gain a foothold and engage in territorial expansion [28]. Inevitably, a turf war ensues when their cellular paths are crossed. Tissue and circulating trophoblasts hinder cancer and metastasis initiation. Conversely the presence of cancer decreases fetal microchimerism in female patients.

#### A feminine hue offers reproductive advantages

It appears that the cancerous placenta (tumor-host interface) and even the whole cancer assume a feminine hue. Analogous to less stringent X inactivation in the female placenta, cancer features expression of X-linked genes [78]. The X chromosome has been implicated in cancer progression, whereas the loss of the Y chromosome is strongly associated with higher cancer risk and poorer cancer prognosis in males [79]. Moreover, the paternal X-chromosome seems to be more likely to undergo inactivation than is the maternal counterpart.

This feminine proclivity in cancer might offer reproductive advantages. Many trophoblast—associated genes are located on the sex chromosomes [80, 81]. The Octamer-binding transcription factor gene (*OCT*, located on the X chromosome) product is at the crossroads of nutrient regulation and chromatin modification. A female preponderance of its activity might account for more efficient energy storage in the female placenta as opposed to aggressive nutrient extraction from the mother by the male placenta. Moreover, the gene product interacts with core histone proteins and ten eleven translocation family (TET), therefore wielding its influence over the expression of many somatic genes to facilitate better placentation in the female placenta besides orchestrating a stronger immune response to various stimuli.

The female placenta seems to have higher global DNA methylation levels than does the male placenta. Similarly, DNA hypermethylation is a consistent feature in cancer and strongly associated with tumor progression [82]. Many of the affected genes actually involve the HLA system, tumor-associated genes and accessory/co-stimulatory molecules.

#### Metastatic cancer: primordial placenta?

The whole metastatic cancer might function as a primordial placenta. Not only is the metastatic cancer more homogeneous than the primary, it appears to more diffusely express trophoblastic markers [83–85]. The metastatic cancer features a vasculature with a diffuse expression of FSH receptors [86]. Furthermore, metastatic cancer cells are more aggressive and possess far superior seeding capability than cells from the primary [87]. As a trade-off, they seem to have lost some of their proliferative prowess [88, 89]. Hypermethylation of *Kiss*-*1 and NM23 genes* genes in metastatic cancer might account for the unopposed trophoblastic differentiation and concurrent shift of the cellular mechanisms from cell division to invasion/migration (“go or grow” concept) [90].

This skewed differentiation toward trophoblast linage evident in metastatic cancer is in keeping with errant expansion of trophoblasts at the expense of the development of the inner cell mass in partial mole pregnancy. Paralleling the over-expression of X-linked genes (XXX, XXY genotypes) in partial mole pregnancy is the less stringent X inactivation in advanced cancer and metastasis [78]. As with cancer progression, neoplastic trophoblastic proliferation and metastasis are also linked to the loss of the Y chromosome [91].

### Common hallmarks and key parameter and molecules

**Fig. 2:**
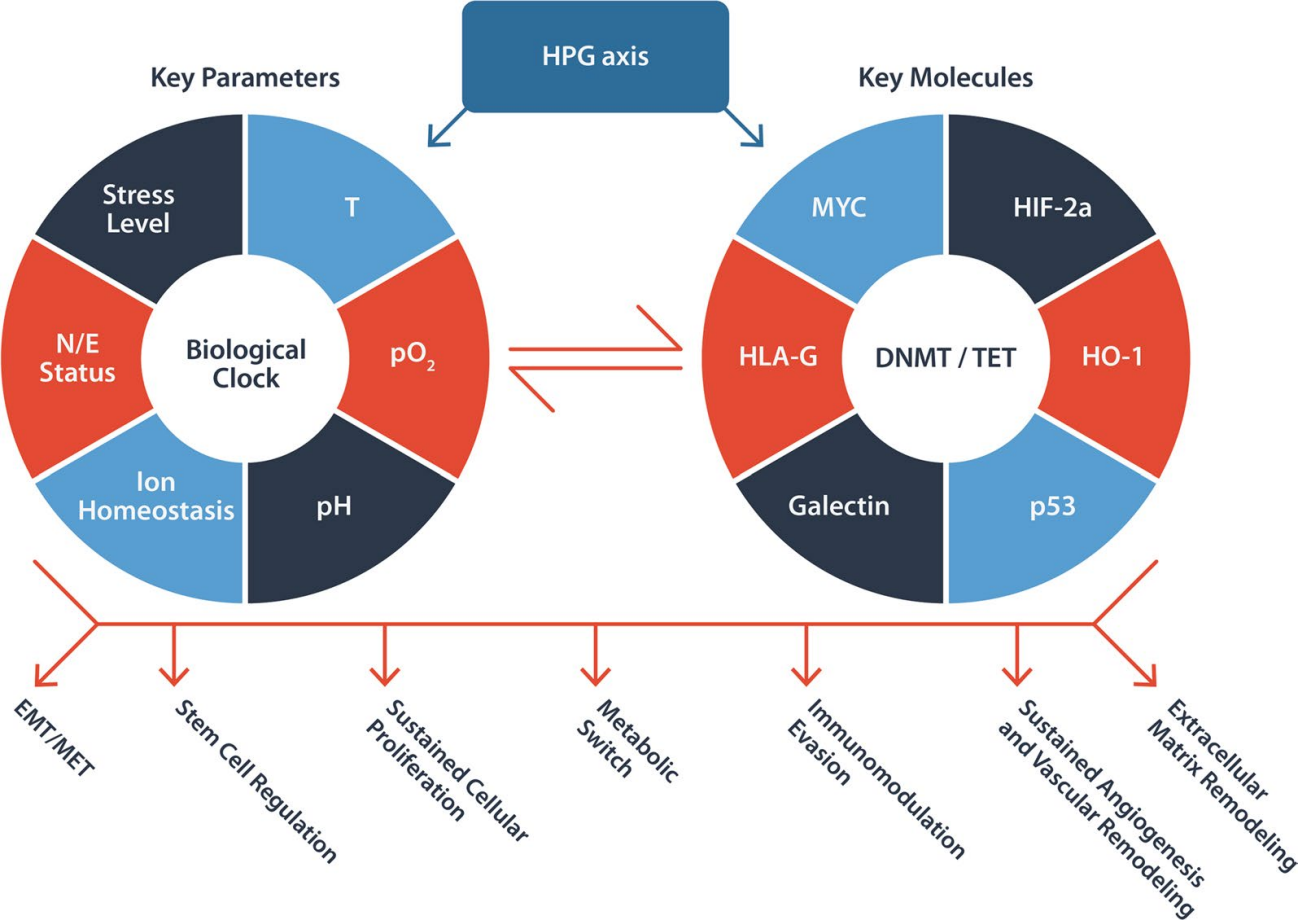
Enactment of the reproductive dramas by the HPG axis via a set of key parameters and small molecules. As each parameter is controlled by a labyrinth of sensing and effecting systems, so is every molecule intricately enmeshed in a network of pathways. These biophysical parameters and biological molecules have coalesced into the bedrock of cellular life through billions years of evolution. DNMT/TET is used to represent the epigenetic regulatory machinery and even microRNAs which are closely interconnected with the machinery. *T* temperature, *p02* partial pressure of oxygen, *pH* power of hydrogen or potential of hydrogen, *N/E* nutrient/energy, *p53* phosphoprotein 53, *HIF* hypoxia inducible factor, *MYC* transcription factor of myelocytomatosis viral oncogene homolog, *HO*-*1* heme oxygenase-1, *HLA*-*G* human leucocyte antigen-G, *DNMT/TET* DNA methyltransferase/ten-eleven translocation methylcytosine dioxygenase, *EMT/MET* epithelial–mesenchymal transition/mesenchymal–epithelial transition

As illustrated in Fig. 2, the manifestation of the shared hallmarks of the two reproductive dramas results from the interpretation and rendering of the script by the HPG axis. The axis execute the renderings via a set of key interacting biological parameters and molecules [92–97]. The biological parameters and molecules have coalesced into the bedrock of cellular life through the co-evolution between life and the biophysical elements on this ever evolving planet.

The axis commands much wider targets and performs much more biological functions than previously thought [32, 98, 99]. More importantly, sex steroid hormones possess the capability of fine- tuning the expression of a same target gene via multiple mechanisms such as direct genomics signaling, indirect genomic signaling, non-genomic signaling [100–102]. In addition to complex interactions among the axis members, some axis hormone receptors can even become activated via a ligand independent mechanism.

### Other parallels

Commensurate to the tripartite relationship in human reproduction (mother–father-embryo), an expedient alliance is forged among the three strange bedfellows (the primary cancer, host and metastases) through coercion and exploitation. In this procreation relationship, the reproductive capacity of the metastatic cancer is suppressed in a manner akin intrauterine suppression of the fetal reproductive development and even to the inhibition of the maternal HPG axis during pregnancy [103, 104]. Presumably due to rising hormone levels during metastasis, metastatic cancer indeed expresses diminished levels of ER, PR compared to the primary cancer [105, 106]. As indirect proof for a negative effect on cancerous ER, PR expression by rising hormone levels during metastasis, large scale epidemiological studies have shown a clear female advantage in cancer survival rates [107, 108]. The advantageous survival rates can be roughly translated into lower metastasis rates or metastasis related deaths. The advantage, however, correlates with the presence of an active HPG axis in the patients and slowly disappears with advancing age.

In addition, similar to the developing placenta, metastatic as well as advanced primary cancer appear to operate on an readjusted cellular clock [109].

## Outlook and ramifications

### Outlook

The metastatic drama bears staggering resemblance to human reproduction. However, before transplanting the pearls of reproductive medicine to cancer research and therapeutics, several pressing issues need to be addressed. The first issue concerns the proper identification and characterization of this small and dynamic population of MICs. It might entail timely combing the primary tumor and bone marrow with a fine-toothed comb. Success might rely on the development of sensitive and specific antibodies and other appropriate detection methods since tumor cells might depend heavily on paracrine signaling thus requiring only low levels of reproductive hormones and receptors. What is more, tumor cells might express receptor variants only and their expression might occur at non-classical subcellular locations.

The second pressing issue is to elucidate the cancer HPG axis and its interactions with the host counterpart. Pioneer work along this line will lay down the framework for efficacious studies targeting the axis for anticancer treatment. In light of the pivotal role of placentation in human reproduction, it is in order that systematic, in-depth studies should be carried out on the tumor/host interface as well as metastatic cancer as a developing placenta. Ultrastructural and molecular studies would not only bear out the placenta concept, but open up a new frontier for cancer research and therapeutics.

### Ramifications

Anticancer therapies aiming at the HPG axis and reproduction -related hormones have been widely reported in cancer research and clinical trials with overall promising results. Inasmuch as there are multiple exquisite and interacting pathways for each and every important cellular function, the efficacy of these therapies could be substantially improved if we target at the whole axis at the same time. The efficacy could be further augmented by the addition of agents and modalities interfering with the downstream cellular parameters and molecules. Many of them have shown anticancer effectiveness individually or in combination. Similarly, as both platelet and bone (marrow) play important roles in both human and cancer reproduction, simple agents such as aspirin and biphosphonates might add additional potency to this anti-reproductive approach [110, 111]. It is thus that we entertain a vision that a day in the near future cancer metastasis can be effectively managed with a regimen formulated on this reproductive theme.

In this reproductive spirit, we are intrigued by the potential of using oral contraceptive pills and even hormone replacement regimens in the treatment and prophylaxis of cancer metastasis [112]. In the same vein, low doses of Metrotrexate might accentuate the effectiveness by aiming at stopping newly colonized MIC clusters in their tracks just as it disrupts ectopic pregnancies. Equally intriguing is the idea of implanting a replaceable subcutaneous patch imbued with molecules rich in the decidua. Such approach would not only thwart the critical step (climax) in metastasis by creating a decoy target for the MICs, but also siphon off cellular and non-cellular resources necessary for the colonization and subsequent development into macrometastases.

Lastly, the proposition of the cancer-host interface as a primitive placenta and the whole metastatic cancer as a primordial placenta might lead to new venues of cancer research and management. It might be more effective, and yet much less detrimental to the patient if we focus our limited resources at disrupting these putatively placental functions such as angiogenesis, nutrient sensing and transportation as well immunomodulation rather than having our sight fixed on destroying the whole primary tumor. Furthermore, the feeder vessels for both the primary and metastasis appear to undergo the same vascular remodeling as do the spiral arteries. Accordingly, tinkering with this important process might lead to breakthroughs in cancer research and treatment.

## Abbreviations

CD56: cluster of differentiation 56
CICs: cancer initiating cells
CXCR4/CYCL12: C–X–C motif chemokine receptor 4/C–X–C Motif Chemokine Ligand 12
DNMT/TET: DNA methyltransferase/ten-eleven translocation methylcytosine dioxygenase
EMT/MET: epithelial–mesenchymal transition/mesenchymal–epithelial transition
ER: estrogen receptor
E-selectin: endothelial selectin
FSH: follicle stimulating hormone
GnRH: gonadotropin-releasing hormone
GPR54: G protein-coupled receptor gene
KiSS1: Kisspeptin-1
HCG: human chorionic gonadotropin
HIF: hypoxia inducible factor
HPG: hypothalamus–pituitary–gonad
HO-1: heme oxygenase-1
HLA: human leukocyte antigen
IGF: insulin-like growth factor
LH: luteinizing hormone
MET: mesenchymal–epithelial transition
MICs: metastasis initiating cells,
MHC: major histocompatibility complex
MAT1: metastasis associated protein 1
MiRNA: microRNA
MYC: transcription factor of myelocytomatosis viral oncogene homolog
NK: natural killer
N/E: nutrient/energy
*OCT*: organic cation transporter
p53: phosphoprotein 53
PARs: platelet associated regulatory system
PAP-A: pregnancy associated protein A
pH: power of hydrogen or potential of hydrogen
p02: partial pressure of oxygen
PGF: placenta growth factor
PIBF: progesterone induced blocking factor
PR: progesterone receptor
PLAP: placental-Like Alkaline Phosphatase
Th1: T helper cell type 1
Th2: T helper cell type 2
T: temperature
VCAM: vascular cell adhesion protein 1 also known as vascular cell adhesion molecule 1
